# Biopolymer: A Sustainable Material for Food and Medical Applications

**DOI:** 10.3390/polym14050983

**Published:** 2022-02-28

**Authors:** Jaya Baranwal, Brajesh Barse, Antonella Fais, Giovanna Lucia Delogu, Amit Kumar

**Affiliations:** 1 DBT-ICGEB Centre for Advanced Bioenergy Research, International Centre for Genetic Engineering & Biotechnology, Aruna Asaf Ali Marg, New Delhi 110067, India; jaya@icgeb.res.in (J.B.); barsebrajesh@icgeb.res.in (B.B.); 2 Department of Life and Environmental Sciences, University of Cagliari, Monserrato, 09042 Cagliari, Italy; fais@unica.it (A.F.); delogug@unica.it (G.L.D.); 3 Department of Electrical and Electronic Engineering, University of Cagliari, Via Marengo 2, 09123 Cagliari, Italy

**Keywords:** biopolymers, medical and food applications, biodegradable materials, microbial polysaccharides, chitosan

## Abstract

Biopolymers are a leading class of functional material suitable for high-value applications and are of great interest to researchers and professionals across various disciplines. Interdisciplinary research is important to understand the basic and applied aspects of biopolymers to address several complex problems associated with good health and well-being. To reduce the environmental impact and dependence on fossil fuels, a lot of effort has gone into replacing synthetic polymers with biodegradable materials, especially those derived from natural resources. In this regard, many types of natural or biopolymers have been developed to meet the needs of ever-expanding applications. These biopolymers are currently used in food applications and are expanding their use in the pharmaceutical and medical industries due to their unique properties. This review focuses on the various uses of biopolymers in the food and medical industry and provides a future outlook for the biopolymer industry.

## 1. Introduction

Biopolymers are the organic substances present in natural sources. The term biopolymer originates from the Greek words bio and polymer, representing nature and living organisms. Large macromolecules made up of numerous repeating units are known as biopolymers [1]. As per the IUPAC definition, a macromolecule defines a single molecule [2]. The biopolymers are found to be biocompatible and biodegradable, making them useful in different applications, such as edible films, emulsions, packaging materials in the food industry, and as drug transport materials, medical implants like medical implants organs, wound healing, tissue scaffolds, and dressing materials in pharmaceutical industries. The main focus of this review is to provide a piece of knowledge about biopolymers and their uses in the food and medical industries.

**Figure 1:**
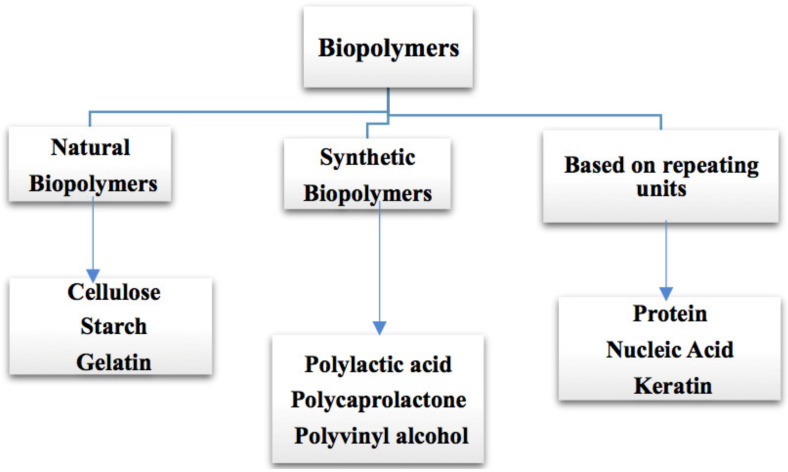
Classification of biopolymers based upon their origin.

The most prevalent macromolecules are biopolymers, which comprise nucleic acids, proteins, carbohydrates, lipids, and giant non-polymeric molecules like lipid and macrocycles, the most frequent macromolecules [3]. Plastics, synthetic fibers, and experimental materials, such as carbon nanotubes, are examples of synthetic macromolecules [4]. In addition to repeating units of nucleic acids, saccharides, or amino acids, their molecular backbones may contain a variety of chemical side chains that contribute to the functions of the molecules. Polylactic acid (PLA) and polyhydroxyalkanoates (PHAs) are two examples of biopolymers found in microorganisms or genetically modified organisms utilizing traditional chemical methods. These include polysaccharides from cellulose and proteins from collagen or milk. The biotechnological synthesis of biopolymers with customized qualities suited for high-value medical applications, such as tissue engineering and medication delivery, is made possible through the genetic modification of microorganisms. According to their origin, the classification of biopolymers is shown in Figure 1, and its advantages, disadvantages are in Table 1. 

### 1.1. Need for Biopolymers

Biopolymers have gotten a lot of interest in various applications where sustainable and biodegradable solutions are needed. The utilization of drug delivery methods to increase the performance of bioactive molecules is still an essential technique for attaining illness therapy, and advancement in this area has been critical. Synthetic, natural, and semi-synthetic polymers are commonly employed in developing drug delivery systems in this context [5]. The standard utilization of synthetic and chemical-based polymers in the food and medical industries has various environmental concerns. Increased environmental awareness of sustainability, controls on pollutants, and municipal solid waste disposal are all driving forces for developing biopolymer-based packaging materials [6]. The use of biopolymers minimizes carbon dioxide emissions, municipal solid waste, and reliance on petroleum-based resources [7].

### 1.2. Sources of Biopolymers

**Figure 2:**
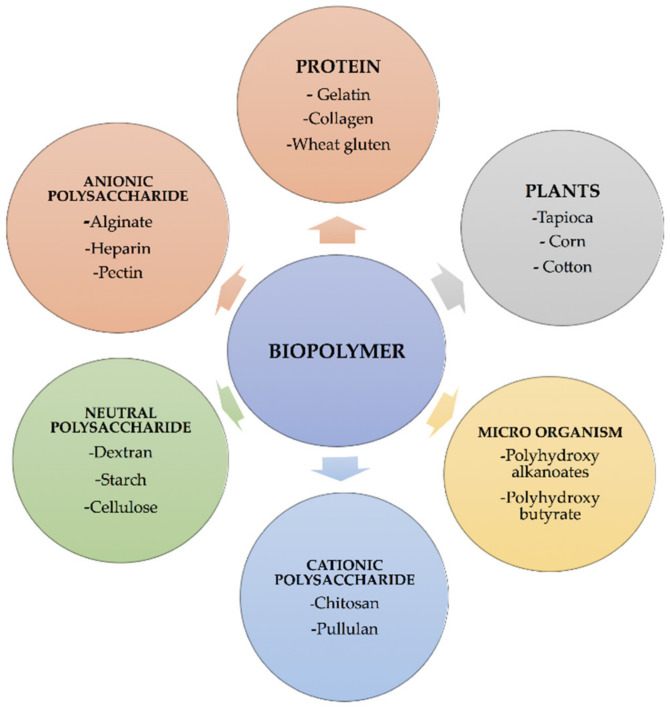
A pictorial depiction of several natural renewable biopolymers categorized according to their source.

**Figure 3:**
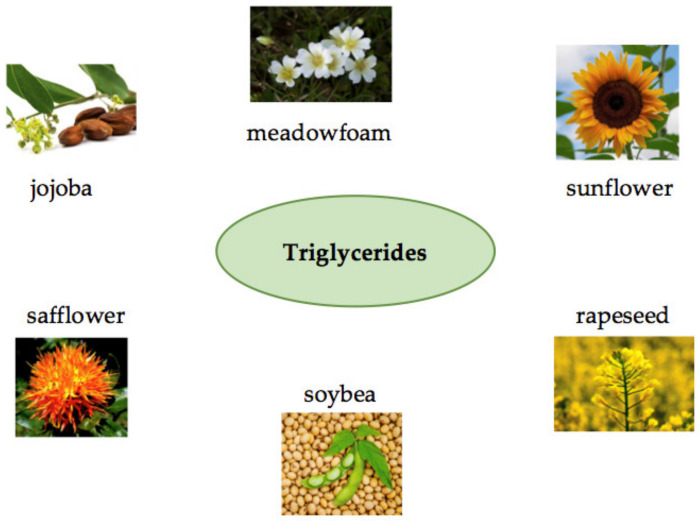
Tryglycerides in vegetable oils are an important source of biopolymers.

Plants, animals, microorganisms, and agricultural wastes are examples of natural biological sources of biopolymers. Plant sources, such as rice, maize [8], wheat [9], sorghum [10], yams [11], cassava [12], potatoes [13], banana [14], tapioca [15], corn [16], cotton [17], and barley [18] biopolymers can be produced chemically from monomeric components, such as oils, sugars, and amino acids. Cattles are the most common animal sources, while corals, sponges, fish, lobster, and shrimp are the most common marine sources. Algae, fungus, and yeasts are the most common microbiological sources (Figure 2). The origins and chemical structures of the main biopolymers are shown in Table 2. Agro leftovers, paper wastes, crops, green wastes, and wood wastes are carbohydrate-rich biomass-based sources. Triglycerides are found in vegetable oils, such as sunflower, soybean, safflower, jojoba, rapeseed, castor, and meadowfoam oil (Figure 3) [19]. Vegetable oils obtained from food producers, in particular, are excellent alternatives for natural polymer synthesis [20]. PHAs are a kind of biopolymer, secondary metabolites generated by microbes and plants. PHAs are stored as inclusion bodies in bacteria and are generated and aggregated intracellularly as transparent granules [21]. These biopolymers are produced naturally and degraded by microbial metabolisms, even though these biopolymers can be melted and shaped in the same way as the chemical and synthetic thermoplastics [22].

#### 1.2.1. As a Bio-Resource: Lignin

**Figure 4:**
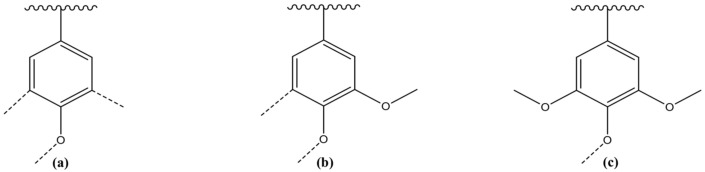
Three structural units in lignin are (**a**) 4-hydroxyphenyl unit, (**b**) guaiacyl unit, and (**c**) syringyl unit.

Lignin is made from renewable resources, including grasses, trees, and plants, and accounts for around 30% of the wood components [32]. Lignins are harmless and have a wide range of applications. The global production of lignin as a by-product of the pulping process exceeds 30 million tons per year. However, it has to be addressed that the mentioned figure is simply an estimate because there are no good statistics on lignin production. After all, it is typically burned as fuel shortly after creation. Lignins for commercial usage are acquired as effluent from the bio-ethanol industry. The lignin higher-order structure, which comprises phenylpropane units, is inherently amorphous. In the process of radical-based lignin biosynthesis, three primary lignin structures, 4-hydroxyphenyl, guaiacyl, and syringyl (Figure 4), are conjugated to generate a three-dimensional lignin polymer [33].

Some natural polymers formed by microbes, plants, or animals alter vital biological information (proteins and nucleic acids). In contrast, there are others (polysaccharides) that offer fuel for cell functions and act as structural components in living systems. Biopolymers can also be synthesized in vitro, which involves treating the biopolymers with isolated enzymes in cell-free settings. One example is using heat-stable DNA polymerases in a polymerase chain reaction (PCR) to create monodisperse specified DNA molecules. A similar example is the manufacture of dextran using isolated dextran sucrose on a smaller scale [34].

#### 1.2.2. Carbohydrate Based Biopolymers: Polysaccharides

Polysaccharides are biopolymers composed of monosaccharides (sugars) connected by glycosidic linkages generated during condensation. The linking of monosaccharides into chains results in radically varying lengths, ranging from two monosaccharides to polysaccharides with thousands of sugars. Polysaccharides serve a variety of essential functions in the biology of living processes [35], and many have found crucial commercial uses. They are split into two classes based on their role in nature: structural and storage polysaccharides. Finally, gums, which are often employed in foods, medicines, and material science emulsifiers, gels, lubricants, adhesives, and other applications, are individually discussed in subsequent sections.

## 2. Biopolymers: State of Art

Humans have primarily utilized biopolymers throughout history as food or in the manufacture of clothes and furnishings. Since the beginning of the Industrial Revolution, fossil fuels, such as oil, have been a crucial energy source for practically every commercial product, including plastic, which is widely used. However, fossil fuels are finite resources, and environmental issues must be considered while using them for both production and energy. We must act sustainably, which means using resources at a rate that allows them to be replenished by our planet’s natural cycles. Biopolymers are experiencing a revival because of their renewable nature. Biopolymers derived from renewable feedstocks have been developed in the last two decades in response to consumer demand for environmentally friendly goods. Polymers made from fossil fuel compete with biopolymers not only for functional qualities but also for cost. Biopolymers are competitive when oil prices are high and feedstock prices, such as maize, starch, are low.

For more than half a century, biochemists and molecular biologists have dominated the research of biopolymers. In recent years, the community of soft matter physics has taken up the biopolymer research area. The goal is not only sheer intellectual curiosity, but also to model and comprehend certain situations. The fundamental physics that underpins biopolymer behavior, and the methodologies used to investigate it, are frequently identical. New powerful X-ray sources have been developed, which has resulted in the creation of new techniques (ultra-high-speed confocal scanning laser microscopy, cryo-TEM), and polymer physicists are currently heavily influenced by single-molecule approaches [36]. There is substantial cooperation among physicists and biologists in this field. Biologists, for example, can develop molecules with specific mutations to explore the function of certain groups put at various points along the sequence, for example, to research the impact of a single residue on the chain. The effect of biopolymer folding–unfolding on mechanical properties may be measured by tugging with an atomic force microscope (AFM) tip [37]. In addition, understanding biological molecular machines enables the creation of synthetic devices [38]. Molecules can conduct similar activities. The observed trend in physics also applies to chemistry, since biopolymers have emerged as novel macromolecular chemistry building blocks in the past decade. This is owing primarily to the introduction of novel polymerization technologies, including controlled free-radical polymerization (CRP) or “click” chemistry. The processing of these chemical alternatives enables precise control of molar mass distribution, macromolecular architecture, and macromolecule functioning. Block copolymers, including one block of a biopolymer, are now more readily available, allowing researchers to examine its bulk self-assembly or a selective solvent for one of the blocks. The biopolymer block’s unique properties in terms of biodegradability, bioactivity, and biocompatibility enable it to be targeted toward fields of application, such as pharmaceutical science, where self-assembly (for example, micellar aggregates, microgels, polymersomes) is utilized to develop drug delivery systems that can be targeted [39]. Furthermore, biopolymer-based block copolymers are particularly promising. The high segregation power demonstrated by the majority of biopolymer blocks compared to synthetic ones results in successful microphase separation, regardless of the modest degrees of polymerization exhibited by each of the blocks individually [40].

Biodegradable polymers have recently gained popularity as an alternative to conventional plastics. Biodegradable packaging is advised, particularly for short-term plastic food packaging, where degradability is a real benefit so that it can be disposed of with food waste for decomposition [41]. In the packaging industry, a variety of natural and synthetic biopolymers are used [42].

## 3. Biopolymers for Medical Applications

Science and technology are essential factors in extending life expectancy. In this regard, various creative approaches and new equipment have been created, resulting in lower morbidity and death rates. The utilization of drug delivery methods to increase the effectiveness of bioactive molecules is an essential technique for treating illnesses, and development in this area has been substantial. Synthetic, semi-synthetic, and natural polymers are commonly employed in the development of drug delivery systems in this context [43]. Suturing, fixing, adhesion, covering, occlusion, isolation, contact inhibition, cell proliferation, tissue guiding, and controlled drug administration [44,45,46,47] are just some of the medicinal applications that biopolymers could be used for. Several biomaterial production techniques and processes have been developed in recent decades to address and examine natural tissues’ important functional architectural and compositional characteristics [48]. The hunt for better and more tissue-oriented implantable units has increased the understanding of biomaterials’ potential and heightened interest in multimodal scaffolds with unique shapes and physical-chemical properties [49]. These scaffolds have multifunctional or multimodal qualities due to the integration of diverse topographies not generally present in each material, which boosts their potential relevance in regenerative medical techniques [50]. Polymers have a critical role in developing three-dimensional templates and the creation of synthetic extracellular matrix (ECM) habitats for tissue regeneration [51]. Biopolymers could be synthesized synthetically or from natural assets [52]. A range of composite materials and interpenetrating networks have been used to attain the required objectives since each group has specific benefits and limits. Due to the adaptability of synthetic polymers, which enables them to be adjusted to a broad range of degradation efficiency, structural traits, and mechanical characteristics, they are a reliable source of innovative materials. The synthetic polymer composition may be tailored to reduce immune response and combine the most significant features. Owing to its superior biodegradability and lack of cytotoxicity, natural polymers have been proposed as a viable alternative to commonly utilized synthetic materials. They are derived from natural sources, resemble soft tissues, and are formed through enzyme-catalyzed chain-growth polymerization processes of activated monomers, often created within cells during metabolic activities. Collagen, gelatin, dextran, agarose/alginate, hyaluronic acid, cellulose, and fibrin gels are all included in this category (Table 3). Despite being cleansed to prevent a foreign body reaction after implantation, natural polymers are commonly employed in regenerative medicine. Biodegradable polymers were chosen for the drug delivery system because they do not need surgery to remove once the medications have been released and may be excreted by the body. Some of the famous examples of biomedical applications that use biopolymers include soft-tissue replacement vascular grafts, breast implants, intraocular lenses, artificial hearts, components of extracorporeal oxygenators, contact lenses, plasmapheresis units, sutures, adhesives, and blood substitutes, dialyzers, liver, pancreas, bladder, kidney, bone cement, catheters, external and internal ear repairs, coatings for pharmaceutical tablets and capsules, cardiac assist devices, implantable pumps, pacemaker, encapsulations, heart valves, artificial blood vessels, joint replacements artificial skin, dentistry, drug delivery, and targeting sites of tumors or inflammation [53,54,55,56]. Since PHAs are biodegradable, they can be used as a substitute for petrochemical plastics in biomedical applications. The adaptability of poly (3-hydroxyoctanoate) in particular makes it a potentially appropriate choice as a biopolymer for drug delivery formulation as well as features of prospective tissue engineering [57].

Many natural biomaterials based on biopolymers have been studied for hard and soft-tissue repair. They can be utilized independently or with other synthetic or inorganic components. The specific dressing, nursing care, and decreased grafting time are the key features of these tissue-engineered materials. Many recent in vivo studies contributed to the FDA clearance of innovative biomaterials for clinical use based on natural biopolymers as matrices for cell distribution and scaffolds for cell-free support of native tissues, despite their mechanical fragility and high cost. For cell encapsulation, a variety of naturally occurring and manufactured biopolymers that might be synthesized into diverse physical forms and geometries are used. The biomaterial component of these therapies must offer suitable mass transport characteristics, membranes, or scaffold stability, and acceptable cellular interactions depending on the site and intended function of the implant. Intelligent biopolymer hydrogels that vary their swelling behavior and other features in response to chemical and physical stimuli, including pH, metabolites or/and ionic variables, temperature, and electric fields, have piqued curiosity. These “smart” hydrogels exhibit single or multiple stimuli-responsive properties. They may be used in a variety of biomedical applications, varying from cell adhesion mediators and controlled drug delivery systems to controllers of gene expression and enzyme function in bioengineering or tissue engineering, in addition to their biocompatibility, biodegradability, and biological processes. Biopolymers may be readily functionalized to improve cell interactions and provide an appropriate platform for cellular and tissue functions. There are two types of peptide-based biopolymers in tissue engineering: self-assembling polypeptides that form gels in response to environmental cues, and polypeptides that create gels by chemical crosslinking. The biopolymer block’s unique bioactivity, biocompatibility, and biodegradability properties enable it to be used in various biomedical applications [70]. Polymer–biopolymer interactions are becoming more and more capable of being planned and selected, allowing for their intervention in cellular dysfunctions and developing more effective, targeted, and efficacious therapeutics. Due to the degree of control possible via live polymerization techniques, previously available structure-function connections for biomacromolecules may now be inferred for fully synthetic materials. Simultaneously, the production and activity of biopolymers may be controlled using molecular biology techniques. Because the ability to control the structure of polymers results in the power to modify their functioning, this synthesis of natural and synthetic macromolecular chemistry inevitably results in biomedical applications. A few examples of the medical application of biopolymers have been presented in Table 3. Bio-printers can automate the assembly process and allow for complex biopolymer manipulation—from the macromolecular to the living cell level—to achieve architectural and biochemical complexity never before possible, resulting in tissue and organ substitutes that closely resemble their natural counterparts. These many regenerative medicine approaches are likely to transition from ‘bench to bedside’ in the future.

The potential of biodegradable nanoparticles has been explored by various researchers for a wide range of biomedical applications due to their properties, such as biocompatibility and bio-safety. Depending on their size, nature, and design, various nanoparticles have distinct therapeutic efficacy, bioavailability, and delivery components. Nanotechnology-assisted imaging is advantageous not only in the field of cancer but also in the field of cardiovascular disease [71,72]. Micelles are helpful in cancer treatment by focusing on malignant cells using tumor molecular probes [73].

### 3.1. Biomedical Applications of Protein-Based Biopolymers

Protein-based biopolymers play a vital role in medical applications. Biopolymers have biocompatible, non-toxic, and biodegradable properties. Their application to implantable devices is expanding rapidly and has great potential [74]. As the molecular sizes of proteins are governed by their secondary structures, the production of nanoparticles proteins permits the formation of precisely constructed nanoparticles [75]. Several types of biodegradable nanoparticles and their characteristics have been summarized in Table 4. Tissue engineering and therapeutic chemical delivery are a few examples of potential uses [76]. Regenerative tissue engineering aims to use the body’s inherent healing potential to improve injured or damaged tissue function. Synthetic polymers and biopolymers are used as scaffolding to establish a favorable environment for cell proliferation and tissue repair. Collagen, keratin, gelatin, sericin, and fibroin are used to make films, pickering emulsions, hydrogels, nanogels, nanofibers, linked porous scaffolds, and 3D-printed scaffolds [47,77,78,79,80,81,82]. Tissue engineering and therapeutics molecules (gene delivery, drug delivery, protein delivery) are delivered via collagen-based nanospheres, nanoparticles, sponges, electrospun fiber, and hydrogel [83]. 

Gelatin is a non-toxic, biodegradable, and FDA-approved substance. Acid (type A gelatin) or essential (type B gelatin) hydrolysis is used to extract from bovine or porcine sources. Gelatin has been extracted from a variety of sources, including jellyfish, fins, bones, and sea urchins [84]. Gel formation, thickening, emulsification, and foaming are characteristics of gelatin [85].

**Table 4:** Various types of biodegradable nanoparticles and their characteristics.

Nanoparticles	Properties	Ref.
Chitosan	Non-toxic, blood viable, antitumor, antioxidant, antimicrobial, inexpensive, and biodegradable	[86]
Superparamagnetic iron oxide nanoparticles	Superparamagnetic, paramagnetic	[87]
Poly-L-lysine	High loading capacity, biodegradable, targeted delivery	[88]
Poly-D-L-lactide-co-glycolide	Biocompatible, non-toxic by-products	[89]
Liposomes	Biocompatible, carries hydrophobic material	[90]
Alginate	Water-soluble, biocompatible	[91]
Gold	Biocompatible, hyperthermia	[92]
Micelles	Capable of carrying water-soluble drug	[93]

### 3.2. Chitosan in Medical Applications

Chitosan is also widely used in medical uses because of its biodegradable and biocompatible properties. It has a variety of uses (gels, films, particles, membranes, or scaffolding, etc.) from biomedical to industrial fields. In addition, due to its properties, chitosan plays an essential role in cell attachment and growth. It is widely used as a tissue engineering matrix when manufactured in a porous structure. The main uses of chitosan in implants are bone, ligament, cartilage, tendon, liver, nerve, stent, and skin regeneration. Several examples of chitosan-based matrices for bone applications have been reported. However, due to its mechanical weakness [94], there is a need to combine chitosan with other materials in applications for bone tissue engineering [95]. Nanoparticles formed using chitosan display interesting properties, such as low toxicity; good absorbability, permeability, and moisture retention; and is easily degradable. However, they suffer from poor long-term stability and are very sensitive to environmental temperature and pH, which affects the degradation rate [96].

## 4. Biopolymers for Food Applications 

In the last 50 years, a growing number of alternative and modified foods have been created using a variety of engineering, science, and biotechnology equipment. As a result, most commercial food on the market today has been altered and enhanced in some way to make it appear better, taste better, and be more financially viable. The advancement of applied engineering and biotechnologies in the food industry has been impressive. From genetic engineering to enhanced preservatives and sophisticated materials for creative materials, food quality management, and packaging, many of the advancements are targeted toward tackling the global food shortage in an ever-increasing population. Although biomedical science has benefited the most from the developments in biopolymer research, recent studies show that new products in biopolymers have a significant influence on food research. These materials are now being researched for novel and unique food items with qualities, such as intelligent detection, better nutritional properties, bio-responsive capabilities, and diverse biodegradable possibilities. Their application is transforming food design, manufacturing, and packaging by supporting an environmentally friendly food engineering strategy. Humans have utilized biopolymers as furniture, clothing, and food since the dawn of time, relying on biomaterials, such as silk, wool, cellulose, and leather. However, these polymers may be customized to meet specific needs in today’s world. Biopolymers derived from natural and contemporary industrial economies contribute significantly to both ecosystems, as humans live in natural and artificial environments. The increasing use of synthetic polymers has created environmental concerns and human health. Materials made from natural polymers are becoming more popular as environmental concerns grow [97].

Food biopolymers are divided into two categories: polysaccharides and proteins. Proteins are amino acid polymers found in plant and animal tissues. Shear, heat, and pH all affect the structural stability of proteins in meals. Polysaccharides, which are branching or linear polymers of sugars bound by glycosidic connections, constitute a significant source of energy in nature. Endo and exo core matrices of marine species and plants include polysaccharides as a hydrated compound. Biopolymers, as part of specialized structures with qualities, are currently widely used in food processing due to their capacity to interact with the other food components to increase their physicochemical properties and stability. Monosaccharides of identical or distinct residues are used to make polysaccharides. They are a visually attractive food packaging platform. Active packaging, composed chiefly of polysaccharide biopolymers, protects food against pathogenic and spoilage germs. Food components undergo phase transformations during preparation and storage at different temperatures and pressures (liquid-gel or liquid-solid). These alterations have an impact on food quality and stability. This is because changes in the physical characteristics of meals are connected to phase transitions in food components [98]. Food phase transition has been emphasized to enhance goods and processes by controlling the process. In recent years, there has been a lot of research on the design and qualities of fat-replaced food items. Biopolymers, particularly hydrocolloids, are often employed to replicate lipids’ sensory and rheological qualities in these food systems.

Biopolymers have been proposed as an appropriate medium for the production and stability of silver nanoparticles [99,100,101]. The polymer-assisted synthesis approach promotes nanoparticle dispersion inside a polymer matrix, which impacts the ultimate structural rigidity and uniformity of the nanocomposite film, resulting in the preservation of significant antibacterial characteristics in nanocomposite films [102,103,104]. The key advantages of nanoparticles are improved food protection and longevity, which may be accomplished by enhancing film-forming materials’ physical and functional characteristics [105].

### 4.1. Nanostructured Coatings on Fruits during COVID-19

Late in 2019, the world was confronted with a new coronavirus (SARS-CoV-2) outbreak in Wuhan, China. COVID-19, the disease caused by SARS-CoV-2, presents a spectrum of moderate-to-severe symptoms, as well as an asymptomatic appearance. The World Health Organization (WHO) proclaimed COVID-19 a pandemic on 11 March 2020, and as of 21 October 2020, over 41 million confirmed cases and 1.13 million fatalities had been documented globally [106]. COVID-19 incidences resulted in millions of infections and deaths worldwide because of its fast spreadability. Like the drugs, healthy fruits with excellent nutritional value have been promoted during the COVID-19 crisis [107]. While the pandemic significantly impacted the economy, it also significantly influenced the healthy supply chain in the forms of fruits from farm to plate due to labor shortages, transportation limitations, and outdoor activities. These were significant factors in spoiling fruits in farms/agricultural areas and developed enormous waste due to limited shelf life. Thus, developing a long-lasting, non-toxic, environmentally friendly, edible nanostructured coatings with antimicrobial properties on the surface of perishable fruits is critical for increasing the fruits’ shelf life and nutritional security.

The edible coating on fruits prevents viral transmission and helps to increase the shelf life of fruits, which is crucial for COVID-19 patient recovery. Polyphenols, a family of phytochemicals having antibacterial, antioxidant, anti-proliferative, and hormone-regulating activities, are found in various fruits, including cranberries, apples, grapes, kiwis, strawberries, blueberries, and citrus fruits [108]. Polyphenols are incredibly beneficial due to their wide variety of health advantages. Polyphenols, such as quercetin, epigallocatechin-gallate, pectolinarin, sinigrin, rhoifolin, gallocatechin-gallate, hesperetin, and herbaceous have been shown in several studies to improve immune function and antiviral activity against coronavirus without generating side effects [109]. It has been noted that coronavirus causes significant lung problems in infected individuals, a condition known as a cytokine storm. The cytokine storm results in an increase in the production of pro-inflammatory cytokines, which contributes to the mortality of infected individuals. Curcumin, emodin, resveratrol, apigenin, kaempferol, and epigallocatechin-gallate have been shown to protect against cytokine storms caused by the coronavirus [110]. Therefore, edible antimicrobial coatings on the fruits are significantly beneficial for preventing virus transmission before infection and extending the postharvest shelf life of polyphenolic fruits, which is critical for immunizing COVID-19 patients during their recovery.

### 4.2. Microbial Polysaccharides in Food Industry

Several polysaccharides are water-soluble gums generated by a diverse range of microorganisms that have innovative and distinctive features. Due to their low cost, these biopolymers have evolved into unique and industrially relevant polymeric compounds. Microbial polysaccharides offer a wide range of uses in the food business due to their structural and physicochemical diversity [111,112]. They are used as stabilizers, emulsifiers, gelling agents, binders, coagulants, and suspending agents. The distinctive rheological qualities of these biopolymers are due to their regular structure and high purity, making them ideal for the food industry. Microbial polysaccharides are non-toxic, biodegradable, and environmentally benign, and they stay active at high temperatures, pH, and salinity. These are suitable substitutes for natural water-soluble gums and synthetics. Due to their superior properties, they may prove to be novel polymers in the food industry as suspending, thickening, and gelling agents [111]. Using genetically engineered microbes under-regulated fermentation settings might lead to the synthesis of new exopolysaccharides with improved characteristics, thus opening up new industrial uses. Polysaccharides can be heteropolymers, or homopolymers, having neutral (pentoses and hexoses) or anionic (uronic acid) sugars, with or without connected non-sugar molecules [113]. As texturizers, thickeners, stabilizers, and gelling agents, microbial polysaccharides improve the flavor, quality, and texture of food [114,115,116].

Sutherland’s tabulation chart of microbial polysaccharide properties and their applications in the food industry is shown in Table 5**.**

### 4.3. The Role of Dietary Fibers in Contemporary Food Production

Traditionally, dietary fibers have been collected from plant sources. However, a recent study has shown that nutritional fibers derived from microbial sources may have comparable benefits. Based on their solubility in water, the dietary fibers are classified as soluble (e.g., gums, β -glucan, pectin, and inulin) or insoluble (e.g., cutin, lignin, suberin, chitin, chitosan, cellulose, hemicellulose, and resistant starches) [129]. Bio-based films are made from a thin matrix of biopolymers with long chains, such as polysaccharides and protein, in a solution or dispersion [130]. To generate a polymer network matrix film, the solvent is removed from the solution using an appropriate approach to reduce the distance between polymers [131]. Bio-based films made from polysaccharides have a wide range of uses in the food business, pharmaceutical sector, cosmetics (anti-aging), and food components [132]. 

Glucans from various natural sources, including plants, algae, yeast, fungus, and bacteria, are helpful to human health because of their physiological effects as biological response modifiers [133], including wound healing benefits [134]. The European Commission included glucan in its list of new food components of functional foods and foods with specific nutritional designations in 2011 [135]. β-glucans also have rheological, biocompatibility, and biodegradability features, making them valuable molecules in various applications. In addition to these unique characteristics, β-glucan can form films, making it a fascinating biopolymer for research in materials science and food contact applications [136].

From diverse sources, several researchers have reported on the possible health effects of β-glucan. The physiological effects of β-glucan are dependent not only on the basis from which it is generated but also on the chemical makeup of the substance [137]. Dietary fiber has been described in various ways over time, and the definition continues to evolve. Dietary fiber’s health advantages are permanent and unaffected by shifting explanations—the chemistry and source of dietary fiber, especially β-glucan, influence these health advantages. Dietary fiber has a wide range of industrial uses due to its health advantages. A larger part of industrial applications relies on some form of extraction. More study is needed to identify the proper dietary fiber intake in youngsters and the elderly. Exploring innovative low-cost extraction processes might lead to new low-cost nutraceutical goods. Overall, nutritional fibers and β-glucan have an enormous potential to drive the nutraceutical industry in the twenty-first century, as customers trust natural ingredients in food to improve their quality of life. 

### 4.4. The Functionality of Starch Derivatives in Bakery and Confectionery Products

Starch is a kind of polysaccharide, which is a long chain of glucose molecules. Starch has two kinds of glucose chains. The first is a basic chain known as amylose, while the second is a complicated branching form known as amylopectin. Starch is the most abundant carbohydrate reserve in plant tubers and seed endosperm, where it is found in the form of granules. In terms of abundance, starch is second only to cellulose [138] as one of the primary carbohydrates synthesized in plants. Starch is also the primary source of energy (60–80%) for many living creatures, including humans. Because of its versatility, it is a very useful component in the food business.

The main component in cookies or bread, especially gluten-free products, is starch, which gives the finished product structure and texture. In the confectionery industry, starches, on the other hand, are mainly used as dusting and gelling agents. Native starches can improve their functional qualities and adaptability under diverse processing circumstances. In the baking and confectionery sectors, starch derivatives were used to replace gluten in gluten-free goods, simulate fat in chocolate fillings, and prevent the bread from staling, among other things. Since starch is a plentiful, affordable, and non-toxic ingredient, interest in using it to produce new food items is very likely to grow. After cellulose, starch is one of the most prevalent carbohydrates in nature. Starch is one of the most studied natural compounds in function and structure [139]. The qualities of individual starch are mainly reliant on its botanical origin, even though it is usually referred to as solitary [140]. Specifically, it is present in high amounts in legumes, cereal grains, tubers, and roots [141] and is found in the tissue of fruits and vegetables [142]. Despite its availability, rice (in Asia), wheat (in the United States and Europe), corn (in the United States and Europe), tapioca (in Brazil, Thailand, and Indonesia), and potato (in Europe) are the only economically sustainable sources of starch [143]. 

Brownie batters, muffin batters, and cake batters are dispersions of sugar, eggs, salt, flour, shortening, leavening agents, and a liquid phase, mainly milk or water. The batter-type dough contains more water than bread dough, which affects how starch behaves and functions in these baked foods. When temperature-triggered in cake-type goods, starch works as a structural agent and transforms a liquid batter into a solid, porous cell structure [144]. Modified starches have become more widely used in biscuits, bakeries, noodles, confectionery, and other convenience foods in recent years. The modified starches application base has grown due to consumers’ desire for healthier and longer-lasting food items. Starches that are modified are used to enhance cake and bread volume, postpone staling of loaves of bread, substitute saturated fats in cookies and confectionery fillings, boost fiber content, and so on. In addition to their conventional role in bakery and confectionery goods, the variety of modified starch used in the food industry is predicted to improve as research in dual and multi-modifier starches to boost starch functionality continues.

### 4.5. Polymer for Food Sector: Guar Gum

Guar gum is a polygalactomannan derived from *Cyamopsis tetragonolobus* endosperm, a leguminous plant. It possesses a backbone of (1-4)-linked beta-D-mannopyranosyl units that contain a single alpha-D-galactopyranosyl team joined every second on the average central chain unit by (1-6) linkage [145]. It produces extraordinarily effective viscosities, even at low concentrations (≤1% *w/v*) in aqueous solutions. Because of these qualities, it is frequently employed as a thickener, foam stabilizer, gelling agent, emulsifier, and antistaling agent in the food industry. Its established prebiotic impact results in decreased blood cholesterol and blood glucose levels. Because of its usage as a dietary fiber, guar gum is also utilized to make foods with a low glycemic index. Furthermore, guar gum has recently been used to produce biodegradable food packaging films and as a wall composition for flavor encapsulation. Paper, textiles, ceramics, mining, cosmetics, paint, explosives, and pharmaceuticals are just a few sectors that use guar gum and its numerous chemical derivatives [146].

Guar, also known as cluster bean (*Cyamopsis tetragonolobus* L.), is a drought-resistant leguminous crop grown primarily in Pakistan and northwest India for use as green fodder, vegetable, and green manure, as well as to extract guar gum from its seeds for industrial use [147]. India produces around 1–1.25 million tons of guar per year, accounting for 80 percent of global guar gum production. Even though India and Pakistan are the world’s leading producers of guar gum, small quantities are also cultivated in the United States, China, Australia, and the African continent. The industrial uses of guar gum account for around 45 percent of total global demand [148]. However, for millennia, the guar plant has been grown in the Indian subcontinent, and the term guar originates from the Sanskrit word gau-ahar, which means “cow” and “food” [149].

Guar gum’s biggest market is the food sector, with several applications (Table 6). By virtue of its unique functionalities, such as reduced evaporation rate, superior water retention capacity, reduced growth of ice crystals, and participation in chemical transformation, it is vital in food applications. Another important characteristic of guar gum that keeps it the preferred hydrocolloid for consumers and businesses is its low cost and natural composition compared to other polymers.

Guar gum is a term that is frequently used in the baking industry to improve baked goods texture, and physical qualities, making gluten-free bread, extending shelf life by minimizing staling, making bread from frozen dough, and as well as a dietary fiber in low glycemic index foods. It is used in various dairy products, including ice creams (to prevent ice crystal growth and improve textural quality), milkshakes (to prevent serum separation and add viscosity and shear resistance), aerated desserts (to gelate and stabilize foam), yogurt (to improve texture and mouthfeel and prevent syneresis), and slimming dietary supplements (for satiation and as a health-promoting nutritional fiber). It has also been used to stabilize yogurt and make low-fat yogurt or products with high dietary fiber content. Lee et al. [157] used guar gum to make yogurt with better rheological properties. Guar gum is a thickening and viscosity enhancer used in the beverage industry. It is soluble in cold water and displays stability at low pH, as seen in drinks, making it a good choice for the beverage sector.

Moreover, because it is an odorless and tasteless molecule, it has no effect on the flavor or taste of the liquid in which it is added. Mostly, guar gum is added to diet beverages in the range of 0.10% to 0.15% (*w*/*v* of beverage) to improve mouthfeel. In fruit juices, it is added in the range of 0.25% to 0.75% (*w*/*v* of juice) to stabilize pulp. Guar gum displays good water-binding capacity and is soluble in hot and cold water. It is primarily employed as a thickening in processed meat products, where it prevents fat migration during storage, provides syneresis control, and regulates rheology and viscosity [158]. It is also used to replace fat in recipes and edible coatings in the meat industry to improve shelf life.

### 4.6. Chitosan Application in Food Industry

After cellulose, chitin, and chitosan are indeed the two most prevalent natural polymers. They can be found in the shells of crustaceans, mollusks, insects, and fungi, but the primary source is the shells of crustaceans. By virtue of chitosan’s unique properties, like biodegradability, nontoxicity, chelating, antioxidant, anticoagulant, antimicrobial, and biocompatible, it has been used to make bioactive materials. Antimicrobial packaging films have been getting more attention from the food industry in recent years [159]. Research into nanofilms made of chitosan polymer has been recently discussed [160]. Chitosans and other marine organisms are drained together during a high-shear cell to make a film that then stops other biomolecules that have been exfoliated. As a result, the water-vapor permeability of the nano-encapsulated chitosan film is significantly reduced, which solves one of the long-running problems in developing biopolymer nanofilms. Moreover, it has been proven that dispersed chitosan can be added to the protein to make nanofilms more durable, making them useful in food preparation. The molecular weight of chitosan mainly determines its properties as a polymer flocculant [161]. Chitosan and its derivative products are natural biopolymers that display antimicrobial [162] antiviral, antifungal, and antioxidant properties [163], with a wide range of applications. Due to the above mentioned beneficial properties, chitosan, and its derivatives have been considered functional food ingredients suitable for people [164].

## 5. Biopolymer Industry

### 5.1. Market Overview

According to an industry analysis research and consulting report on the global polymer market, its size was estimated to be a staggering $666.6 billion in 2018. The market is expected to grow significantly at a compound annual growth rate (CAGR) of 5.1%. The global polymer market is heavily disrupted by biopolymers, one of the most important organic compounds in the world. The classification of biopolymers depends primarily on their end-use industry. This market enjoys a diverse number of end-users, including promising ones in the pharmaceutical, healthcare, food, and beverage industries. In the medical sector, biodegradable polyester is very useful for manufacturing surgical implants. In the food and beverage industry, biopolymers are mainly used in the production of cellophane films and are widely used in food packaging. In 2018, the global biopolymer market showed significant growth; the estimated size was $12 billion. It is expected that during the 2019–2025 period, the biopolymer market will grow significantly at a CAGR of 19%.

### 5.2. Market Outlook

Biopolymers are simple macromolecules made from organisms that are fully biodegradable. Common examples of biopolymers include carbohydrates, proteins, DNA, RNA, nucleic acids, lipids, and peptides; since they are made from living organisms, they are totally carbon neutral and can be easily recycled or renewed. Moreover, biopolymers absorb the carbon dioxide plants emit instead of released into the atmosphere. There are four types of biopolymers: sugar-based biopolymers, synthetic biopolymers, cellulosic biopolymers, and natural polymers. The European Biomass Industry Association has made several efforts to boost the use of biopolymers in the market, as reflected in Europe’s 55% share of the global market in 2018 [165]. 

### 5.3. Major Growth Drivers

Colin Campbell, the co-founder of the London-based Oil Depletion Analysis Center, found with his research work that around 944 billion barrels of oil had been produced in human history, and only 746 billion reserves have not yet been mined [166]. These statistics are alarming, as it is not far from a time when people are utterly dependent on an oil-free world. The depletion of oil reserves is giving the global biopolymer market a vital boost, as companies are forced to innovate, invest and invent. 

Biopolymers are widely used in the pharmaceutical industry to heal wounds of any shape, size, or depth. Some common biopolymers, such as chitosan, gelatin, alginate, and pectin are used to produce hydrogels to help create a moist environment for dry wounds. These biopolymers are also utilized in the manufacture of wound dressings. Altogether, these factors serve as the estimated growth driver for the global biopolymer market. The initial expenditure required to create a product is vital in the biopolymer business. Key market participants are attempting to solve this issue by forming a joint venture with an agriculture firm to build a symbiotic connection for the biopolymer’s growth.

Several major market players are active in the biopolymer market, including ASF SE, Danimer Scientific, Novamont SpA, Galatea Bio Tech, Total Corbion, Plantic Technologies Ltd., FMC BioPolymer AS, NatureWorks LLC, Sigma-Aldrich, and Biome Technologies Ltd. Sigma-Aldrich is a Missouri-based biotechnology and polymer manufacturer. They have developed a variety of natural and biopolymers, such as adhesive carbohydrates, proteins, starch, cellulose, gelatin, chitosan, lignin dextran, collagen, and polyamino acids.

## 6. Conclusions and Future Perspectives

Industrial interest in biopolymer has steadily increased over the decades. The demand for new materials from future manufacturers of biopolymers is overwhelming. However, the material’s cost-effectiveness needs to improve as it is explicitly made available for sustainable development. Bio-based polymers are closer than ever to traditional polymers. Today, with advanced research and development in biotechnology and public awareness, bio-based polymers are commonly found in various applications, from consumer goods to high-tech applications. Food packaging plays an essential role in protecting food from external contamination and maintaining quality, integrity, and safety throughout its shelf life. Materials based on synthetic polymers are used primarily as packaging materials in the food industry due to their ease of manufacture, versatility, affordability, functionality, lightweight, flexibility, and low cost. However, these synthetic polymers are not degradable, and most plastic scraps and debris pollute the environment badly. This requires the development and use of biodegradable polymer materials to solve these environmental problems. Biopolymers or renewable resource-based biopolymers include carboxymethyl cellulose, hemicellulose, pectins, carboxymethyl cellulose, starch, xanthan gum, pullulan, etc. Alginate, guar gum, gum karaya, agar, and gellan, etc., have great potential to replace traditional petroleum-based food packaging materials. The use of biopolymers, such as PLA silk and chitosan are increasingly being explored for medicine applications. The unique properties of biopolymers, such as biocompatibility and biodegradability, offer significant benefits and increase their potential use in implantable medical applications. These new materials are of great importance in medicine, as synthetic materials do not meet the requirements of biological systems. Thus, recent research has demonstrated that using biopolymers in combination with synthetic materials can revolutionize medicine.

Demand for biodegradable polymers is increasing due to environmental concerns about the use of non-renewable materials. Polystyrene and other plastics are some of the most commonly manufactured materials for packaging and other benefits. Such substances cause land and water pollution and have been associated with human and animal health problems in some studies. To solve these problems, scientists, and engineers are enthusiastically innovating to create new biodegradable polymers. They are widely used in various fields and applications, such as packaging, agriculture, and healthcare. The various types of materials used in the development of biodegradable polymers have been researched and tested by scientists and engineers to evaluate their efficacy, safety, and environmental impact. Biodegradable polymers are becoming the standard for plastic packaging that is integrated into society and promotes a healthy and sustainable lifestyle. In fact, it is becoming the next big thing. The biodegradable plastics market is expected to grow to $6.12 billion by 2023, according to the Markets and Markets annual report [167].

Bio-based polymers are currently in the research and development phase to replace existing polymers. Researchers are looking for new materials that can be made to offset the use of petroleum-based polymers. Bio-based polymers are composed of renewable raw materials. Currently, these polymers make up a small portion (less than 1%) of the plastics market. Bio-based polymers are formulated via a bacterial fermentation process that synthesizes monomers from renewable resources from agricultural plants, fatty acids, lignocellulosic biomass, and organic waste. Natural bio-based polymers are inherently found in various proteins (such as collagen) and nucleic acids. The latest breakthrough expected in the plastics industry is to make plastics more biodegradable while maintaining strength and durability that compete with ordinary plastics.

## Figures and Tables

**Table 1:** Principal advantages and disadvantages of biopolymers.

Biopolymers	Advantages	Disadvantages	Reference
Natural Biopolymers	Biologically renewable, biodegradable, biocompatible, non-toxic, bioadhesive material, biofuctional.	Less stable, low melting point, high surface tension, structurally more complex.	[2]
Synthetic Biopolymers	Biocompatibility, higher reproducibility, better mechanical, and chemical stability	Toxic, non-biodegradable, expensive synthesis procedure.	[4]

**Table 2:** Main biopolymers with their origins and chemical structures [23].

Biopolymers	Sources	Structure	Reference (Ref.)
Chitin	Corals, horseshoe worms, lamp shells, sponges, squid, cuttlefish, and clams are examples of aquatic species	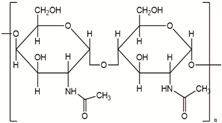	[24,25]
Chitosan	Fungi, mollusks, algae, crustaceans, and insects	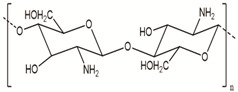	[24,26]
Cellulose	Agricultural trashes, such as Seaweed, rice husk, and sugarcane bagasse. Plant sources like wood, bamboo, sugarbeet, banana rachis, potato tubers, cotton, fique, kapok, agave, jute, kenaf, flax, hemp, vine, sisal, coconut, grass, wheat, rice, and barley	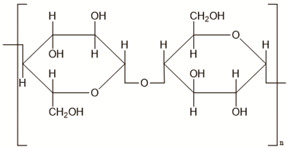	[27]
Alginate	Seawood	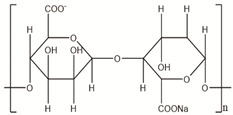	[28]
Starch	Potatoes, maize, cassava, rice, sorghum, banana wheat, yams	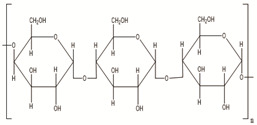	[29]
Cyclodextrin	Starch sources like tapioca, potato, wheat, rice, and corn	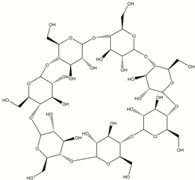	[30]
Polycaprolactone	Polycondensation of **ε** -caprolactone	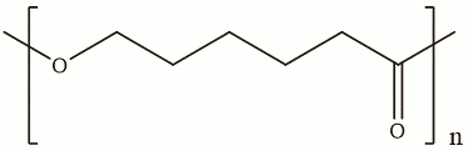	[31]

**Table 3:** Examples of some biopolymers with their medical application.

Biopolymer	Medical Application	Ref.
Collagen	Surface coating for tissue culture plates	[58]
	Simple gels for cell culture	
Alginate	Regenerative medicine	[59]
	Tissue engineering	
Hyaluronic acid	Treatment and lubrication of damaged joints	[60]
	Cutaneous and corneal wound healing	
Fibrin	Blood clotting, wound healing, and tumor growth	[61]
	Hemostatic agent, sealant, and surgical glue	
Silk fibroin	Regenerative medicine Treatment of wounds, bioengineering of tissues	[62]
Agarose	Skeletal tissues regeneration, kidney and fibroblast encapsulation	[63]
Carrageenan	Skeletal tissues regeneration, cell delivery system	[64]
Fibronectin	Wound healing, cardiac repair, bone regeneration	[65]
PHAs	Drug delivery systems, one tissue regeneration,	[66]
Elastin	soft-tissue reconstruction, orthopedics and cell encapsulation	[67]
Keratin	Cornea tissue engineering, skin regeneration	[68]
Starch	Bone and cartilage regeneration, spinal cord injury treatment	[69]

**Table 5:** Properties and applications of microbial polysaccharides.

Biopolymers	Properties	Applications	Ref.
Carboxymethyl- cellulose	Coating, Emulsifying agent	Confectionary Salad dressing	[117] [118]
Hemicellulose	Binding agent	Pet foods	[119]
Pectins	Adhesive	Icings and glazes	[120]
Starch	Stabilizer	Ice cream, salad dressing	[121]
Xanthan gum	Foam stabilizer	Beer	[122]
Pullulan	Film formation	Protective coating	[123]
Alginate	Gelling agent	Confectionary milk-based desserts, jellies	[124]
Guar gum	Thickening agent	Jams, syrups, and pie fillings	[125]
Gum karaya	Syneresis inhibitor	Frozen foods, cheeses	[126]
Agar	Swelling agent	Processed meat products	[127]
Gellan	Inhibitor	Frozen foods, sugar syrups	[128]

**Table 6:** Guar Gum’s Various Applications in the Food Industry.

Properties	Applications	Ref.
Improving Textures	Stabilizer, thickener, gluten-free noodles, emulsifier, reducing oil uptake during fry	[150]
Beverage Industry	Thickener, stabilizer, dietary fiber	[151]
Dairy Products	Viscosifier, improving texture and mouthfeel, foam stabilization, preventing ice crystal growth in ice creams	[152]
Meat Products	Edible films, fat replacer, thickener	[153]
Soluble type of dietary fiber	Prebiotic, reducing blood, sugar, and cholesterol, treating constipation and diarrhea	[154]
Bakery industry	Frozen dough improvement, gluten-free products, texture, and physical property improvement	[155]
Others	Biodegradable films, flavor encapsulation	[156]

## Data Availability

In Data is contained within the article.

## References

[1] Ezeoha S.L. (2013). Production of Biodegradable Plastic Packaging Film from Cassava Starch. IOSR J. Eng..

[2] Jenkins A.D., Kratochvíl P., Stepto R.F.T., Suter U.W. (1996). Glossary of basic terms in polymer science (IUPAC Recommendations 1996). Pure Appl. Chem..

[3] Stryer L., Berg J.M., Tymoczko J.L. (2002). Biochemistry And Study Guide.

[4] Gullapalli S., Wong M.S. (2011). Nanotechnology: A Guide to Nano-Objects. Chem. Eng. Prog..

[5] Ruso J.M., Messina P.V. (2017). Biopolymers for Medical Applications.

[6] Siracusa V., Rocculi P., Romani S., Rosa M.D. (2008). Biodegradable polymers for food packaging: A review. Trends Food Sci. Technol..

[7] Peelman N., Ragaert P., De Meulenaer B., Adons D., Peeters R., Cardon L., Van Impe F., Devlieghere F. (2013). Application of bioplastics for food packaging. Trends Food Sci. Technol..

[8] Awad Y.M., Blagodatskaya E., Ok Y.S., Kuzyakov Y. (2013). Effects of polyacrylamide, biopolymer and biochar on the decomposition of14C-labelled maize residues and on their stabilization in soil aggregates. Eur. J. Soil Sci..

[9] Karmanov A.P., Kanarsky A.V., Kocheva L.S., Belyy V.A., Semenov E.I., Rachkova N.G., Bogdanovich N.I., Pokryshkin S.A. (2021). Chemical structure and polymer properties of wheat and cabbage lignins–Valuable biopolymers for biomedical applications. Polymer.

[10] Tanamool V., Imai T., Danvirutai P., Kaewkannetra P. (2013). An alternative approach to the fermentation of sweet sorghum juice into biopolymer of poly-β-hydroxyalkanoates (PHAs) by newly isolated, Bacillus aryabhattai PKV01. Biotechnol. Bioprocess Eng..

[11] Gutiérrez T.J., Pérez E., Guzmán R., Tapia M.S., Famá L. (2014). Physicochemical and Functional Properties of Native and Modified by Crosslinking, Dark-Cush-Cush Yam (*Dioscorea trifida*) and Cassava (*Manihot esculenta*) Starch. J. Polym. Biopolym. Phys. Chem..

[12] Perotti G.F., Tronto J., Bizeto M.A., Izumi C.M.S., Temperini M.L.A., Lugão A.B., Parra D.F., Constantino V.R.L. (2013). Biopolymer-Clay Nanocomposites: Cassava Starch and Synthetic Clay Cast Films. J. Braz. Chem. Soc..

[13] Borah P.P., Das P., Badwaik L.S. (2017). Ultrasound treated potato peel and sweet lime pomace based biopolymer film development. Ultrason. Sonochem..

[14] Redondo-Gómez C., Rodríguez Quesada M., Vallejo Astúa S., Murillo Zamora J.P., Lopretti M., Vega-Baudrit J.R. (2020). Biorefinery of Biomass of Agro-Industrial Banana Waste to Obtain High-Value Biopolymers. Molecules.

[15] Abral H., Dalimunthe M.H., Hartono J., Efendi R.P., Asrofi M., Sugiarti E., Sapuan S.M., Park J.-W., Kim H.-J. (2018). Characterization of Tapioca Starch Biopolymer Composites Reinforced with Micro Scale Water Hyacinth Fibers. Starch-Stärke.

[16] Lauer M.K., Tennyson A.G., Smith R.C. (2021). Inverse vulcanization of octenyl succinate-modified corn starch as a route to biopolymer–sulfur composites. Mater. Adv..

[17] Aguilar N.M., Arteaga-Cardona F., de Anda Reyes M.E., Gervacio-Arciniega J.J., Salazar-Kuri U. (2019). Magnetic bioplastics based on isolated cellulose from cotton and sugarcane bagasse. Mater. Chem. Phys..

[18] Yu P. (2007). Molecular chemical structure of barley proteins revealed by ultra-spatially resolved synchrotron light sourced FTIR microspectroscopy: Comparison of barley varieties. Biopolymers.

[19] Flaris V., Singh G. (2009). Recent developments in biopolymers. J. Vinyl Addit. Technol..

[20] Sharma V., Kundu P.P. (2006). Addition polymers from natural oils—A review. Prog. Polym. Sci..

[21] Muhamad I.I., Sabbagh F., Abdul Karim N. (2017). Polyhydroxyalkanoates: A Valuable Secondary Metabolite Produced in Microorganisms and Plants. Plant Secondary Metabolites.

[22] Mojaveryazdi F.S., Muhamad I.I., Rezania S., Benham H. (2014). Importance of Glucose and Pseudomonas in Producing Degradable Plastics. J. Teknol..

[23] Udayakumar G.P., Muthusamy S., Selvaganesh B., Sivarajasekar N., Rambabu K., Sivamani S., Sivakumar N., Maran J.P., Hosseini-Bandegharaei A. (2021). Ecofriendly biopolymers and composites: Preparation and their applications in water-treatment. Biotechnol. Adv..

[24] Elieh-Ali-Komi D., Hamblin M.R. (2016). Chitin and Chitosan: Production and Application of Versatile Biomedical Nanomaterials. Int. J. Adv. Res..

[25] Satitsri S., Muanprasat C. (2020). Chitin and Chitosan Derivatives as Biomaterial Resources for Biological and Biomedical Applications. Molecules.

[26] Aranaz I., Alcántara A.R., Civera M.C., Arias C., Elorza B., Heras Caballero A., Acosta N. (2021). Chitosan: An Overview of Its Properties and Applications. Polymers.

[27] Farooq A., Patoary M.K., Zhang M., Mussana H., Li M., Naeem M.A., Mushtaq M., Farooq A., Liu L. (2020). Cellulose from sources to nanocellulose and an overview of synthesis and properties of nanocellulose/zinc oxide nanocomposite materials. Int. J. Biol. Macromol..

[28] Lee K.Y., Mooney D.J. (2012). Alginate: Properties and biomedical applications. Prog. Polym. Sci..

[29] Carvalho A.J.F. (2008). Starch: Major Sources, Properties and Applications as Thermoplastic Materials. Monomers, Polymers and Composites from Renewable Resources.

[30] Li Z., Wang M., Wang F., Gu Z., Du G., Wu J., Chen J. (2007). γ-Cyclodextrin: A review on enzymatic production and applications. Appl. Microbiol. Biotechnol..

[31] Espinoza S.M., Patil H.I., San Martin Martinez E., Casañas Pimentel R., Ige P.P. (2019). Poly-ε-caprolactone (PCL), a promising polymer for pharmaceutical and biomedical applications: Focus on nanomedicine in cancer. Int. J. Polym. Mater. Polym. Biomater..

[32] Hatakeyama H., Hatakeyama T. (2009). Lignin Structure, Properties, and Applications. Biopolymers.

[33] Donaldson L., Hague J., Snell R. (2001). Lignin Distribution in Coppice Poplar, Linseed and Wheat Straw. Holzforschung.

[34] Kato Y., Onishi H., Machida Y. (2003). Application of Chitin and Chitosan Derivatives in the Pharmaceutical Field. Curr. Pharm. Biotechnol..

[35] Gangalla R., Gattu S., Palaniappan S., Ahamed M., Macha B., Thampu R.K., Fais A., Cincotti A., Gatto G., Dama M. (2021). Structural Characterisation and Assessment of the Novel Bacillus amyloliquefaciens RK3 Exopolysaccharide on the Improvement of Cognitive Function in Alzheimer’s Disease Mice. Polymers.

[36] Deniz A.A., Mukhopadhyay S., Lemke E.A. (2007). Single-molecule biophysics: At the interface of biology, physics and chemistry. J. R. Soc. Interface.

[37] Alessandrini A., Facci P. (2005). AFM: A versatile tool in biophysics. Meas. Sci. Technol..

[38] Kay E.R., Leigh D.A., Zerbetto F. (2007). Synthetic Molecular Motors and Mechanical Machines. Angew. Chem. Int. Ed..

[39] Schatz C., Lecommandoux S. (2010). Polysaccharide-Containing Block Copolymers: Synthesis, Properties and Applications of an Emerging Family of Glycoconjugates. Macromol. Rapid Commun..

[40] Cushen J.D., Otsuka I., Bates C.M., Halila S., Fort S., Rochas C., Easley J.A., Rausch E.L., Thio A., Borsali R. (2012). Oligosaccharide/Silicon-Containing Block Copolymers with 5 nm Features for Lithographic Applications. ACS Nano.

[41] Song J.H., Murphy R.J., Narayan R., Davies G.B.H. (2009). Biodegradable and compostable alternatives to conventional plastics. Philos. Trans. R. Soc. B Biol. Sci..

[42] Miles C., DeVetter L., Ghimire S., Hayes D.G. (2017). Suitability of Biodegradable Plastic Mulches for Organic and Sustainable Agricultural Production Systems. HortScience.

[43] Gutierrez Cisneros C., Bloemen V., Mignon A. (2021). Synthetic, Natural, and Semisynthetic Polymer Carriers for Controlled Nitric Oxide Release in Dermal Applications: A Review. Polymers.

[44] Neuendorf R.E., Saiz E., Tomsia A.P., Ritchie R.O. (2008). Adhesion between biodegradable polymers and hydroxyapatite: Relevance to synthetic bone-like materials and tissue engineering scaffolds. Acta Biomater..

[45] Chen Y., Hung S.-T., Chou E., Wu H.-S. (2018). Review of Polyhydroxyalkanoates Materials and other Biopolymers for Medical Applications. Mini Rev. Org. Chem..

[46] Tsai C.-H., Wang P.-Y., Lin I.C., Huang H., Liu G.-S., Tseng C.-L. (2018). Ocular Drug Delivery: Role of Degradable Polymeric Nanocarriers for Ophthalmic Application. Int. J. Mol. Sci..

[47] Park S.-B., Lih E., Park K.-S., Joung Y.K., Han D.K. (2017). Biopolymer-based functional composites for medical applications. Prog. Polym. Sci..

[48] Yahya E.B., Amirul A.A., Khalil H.P.S.A., Olaiya N.G., Iqbal M.O., Jummaat F., Atty Sofea A.K., Adnan A.S. (2021). Insights into the Role of Biopolymer Aerogel Scaffolds in Tissue Engineering and Regenerative Medicine. Polymers.

[49] Chaikof E.L., Matthew H., Kohn J., Mikos A.G., Prestwich G.D., Yip C.M. (2002). Biomaterials and Scaffolds in Reparative Medicine. Ann. N. Y. Acad. Sci..

[50] Song W., Lima A.C., Mano J.F. (2010). Bioinspired methodology to fabricate hydrogel spheres for multi-applications using superhydrophobic substrates. Soft Matter.

[51] Hook A.L., Anderson D.G., Langer R., Williams P., Davies M.C., Alexander M.R. (2010). High throughput methods applied in biomaterial development and discovery. Biomaterials.

[52] Hassan N., Verdinelli V., Ruso J.M., Messina P.V. (2012). Assessing structure and dynamics of fibrinogen films on silicon nanofibers: Towards hemocompatibility devices. Soft Matter.

[53] Pattanashetti N.A., Heggannavar G.B., Kariduraganavar M.Y. (2017). Smart Biopolymers and their Biomedical Applications. Procedia Manuf..

[54] Reddy N., Reddy R., Jiang Q. (2015). Crosslinking biopolymers for biomedical applications. Trends Biotechnol..

[55] Wróblewska-Krepsztul J., Rydzkowski T., Michalska-Pożoga I., Thakur V.K. (2019). Biopolymers for Biomedical and Pharmaceutical Applications: Recent Advances and Overview of Alginate Electrospinning. Nanomaterials.

[56] Gagliardi A., Giuliano E., Venkateswararao E., Fresta M., Bulotta S., Awasthi V., Cosco D. (2021). Biodegradable Polymeric Nanoparticles for Drug Delivery to Solid Tumors. Front. Pharmacol..

[57] Sabbagh F., Muhamad I.I. (2017). Production of poly-hydroxyalkanoate as secondary metabolite with main focus on sustainable energy. Renew. Sustain. Energy Rev..

[58] Tripathi D., Rastogi K., Tyagi P., Rawat H., Mittal G., Jamini A., Singh H., Tyagi A. (2021). Comparative Analysis of Collagen and Chitosan-based Dressing for Haemostatic and Wound Healing Application. AAPS PharmSciTech.

[59] Álvarez-Castillo E., Aguilar J.M., Bengoechea C., López-Castejón M.L., Guerrero A. (2021). Rheology and Water Absorption Properties of Alginate–Soy Protein Composites. Polymers.

[60] Dulińska-Litewka J., Dykas K., Felkle D., Karnas K., Khachatryan G., Karewicz A. (2021). Hyaluronic Acid-Silver Nanocomposites and Their Biomedical Applications: A Review. Materials.

[61] Nelson D.W., Gilbert R.J. (2021). Extracellular Matrix-Mimetic Hydrogels for Treating Neural Tissue Injury: A Focus on Fibrin, Hyaluronic Acid, and Elastin-Like Polypeptide Hydrogels. Adv. Healthc. Mater..

[62] Grabska-Zielińska S., Sionkowska A. (2021). How to Improve Physico-Chemical Properties of Silk Fibroin Materials for Biomedical Applications?—Blending and Cross-Linking of Silk Fibroin—A Review. Materials.

[63] Mahmood A., Patel D., Hickson B., DesRochers J., Hu X. (2022). Recent Progress in Biopolymer-Based Hydrogel Materials for Biomedical Applications. Int. J. Mol. Sci..

[64] Khaliq T., Sohail M., Minhas M.U., Ahmed Shah S., Jabeen N., Khan S., Hussain Z., Mahmood A., Kousar M., Rashid H. (2022). Self-crosslinked chitosan/κ-carrageenan-based biomimetic membranes to combat diabetic burn wound infections. Int. J. Biol. Macromol..

[65] Klavert J., van der Eerden B.C.J. (2021). Fibronectin in Fracture Healing: Biological Mechanisms and Regenerative Avenues. Front. Bioeng. Biotechnol..

[66] Goswami M., Rekhi P., Debnath M., Ramakrishna S. (2021). Microbial Polyhydroxyalkanoates Granules: An Approach Targeting Biopolymer for Medical Applications and Developing Bone Scaffolds. Molecules.

[67] Sarangthem V., Singh T.D., Dinda A.K. (2021). Emerging Role of Elastin-Like Polypeptides in Regenerative Medicine. Adv. Wound Care.

[68] Sellappan L.K., Anandhavelu S., Doble M., Perumal G., Jeon J.-H., Vikraman D., Kim H.-S. (2020). Biopolymer film fabrication for skin mimetic tissue regenerative wound dressing applications. Int. J. Polym. Mater. Polym. Biomater..

[69] Varma K., Gopi S. (2021). Biopolymers and their role in medicinal and pharmaceutical applications. Biopolymers and Their Industrial Applications.

[70] Yadav P. (2015). Biomedical Biopolymers, their Origin and Evolution in Biomedical Sciences: A Systematic Review. J. Clin. Diagn. Res..

[71] Bhatia M., Girdhar A., Tiwari A., Nayarisseri A. (2014). Implications of a novel Pseudomonas species on low density polyethylene biodegradation: An in vitro to in silico approach. SpringerPlus.

[72] Liu C., Li Z., Du B., Han C., Hou Z. Effect of Fullerene Nanoparticle on Tuning Trap Level Distribution of Fullerene/Polyethylene Nanocomposites. Proceedings of the 2019 2nd International Conference on Electrical Materials and Power Equipment (ICEMPE).

[73] Peters D., Kastantin M., Kotamraju V.R., Karmali P.P., Gujraty K., Tirrell M., Ruoslahti E. (2009). Targeting atherosclerosis by using modular, multifunctional micelles. Proc. Natl. Acad. Sci. USA.

[74] Rebelo R., Fernandes M., Fangueiro R. (2017). Biopolymers in Medical Implants: A Brief Review. Procedia Eng..

[75] Nitta S., Numata K. (2013). Biopolymer-Based Nanoparticles for Drug/Gene Delivery and Tissue Engineering. Int. J. Mol. Sci..

[76] Nagarajan S., Radhakrishnan S., Kalkura S.N., Balme S., Miele P., Bechelany M. (2019). Overview of Protein-Based Biopolymers for Biomedical Application. Macromol. Chem. Phys..

[77] Nagarajan S., Abessolo Ondo D., Gassara S., Bechelany M., Balme S., Miele P., Kalkura N., Pochat-Bohatier C. (2018). Porous Gelatin Membrane Obtained from Pickering Emulsions Stabilized by Graphene Oxide. Langmuir.

[78] Biscarat J., Bechelany M., Pochat-Bohatier C., Miele P. (2015). Graphene-like BN/gelatin nanobiocomposites for gas barrier applications. Nanoscale.

[79] Li Y., Rodrigues J., Tomás H. (2012). Injectable and biodegradable hydrogels: Gelation, biodegradation and biomedical applications. Chem. Soc. Rev..

[80] Kharkar P.M., Kiick K.L., Kloxin A.M. (2013). Designing degradable hydrogels for orthogonal control of cell microenvironments. Chem. Soc. Rev..

[81] Sridhar R., Lakshminarayanan R., Madhaiyan K., Amutha Barathi V., Lim K.H.C., Ramakrishna S. (2015). Electrosprayed nanoparticles and electrospun nanofibers based on natural materials: Applications in tissue regeneration, drug delivery and pharmaceuticals. Chem. Soc. Rev..

[82] Jammalamadaka U., Tappa K. (2018). Recent Advances in Biomaterials for 3D Printing and Tissue Engineering. J. Funct. Biomater..

[83] Lee C.H., Singla A., Lee Y. (2001). Biomedical applications of collagen. Int. J. Pharm..

[84] Gómez-Guillén M.C., Giménez B., López-Caballero M.E., Montero M.P. (2011). Functional and bioactive properties of collagen and gelatin from alternative sources: A review. Food Hydrocoll..

[85] Gaspar-Pintiliescu A., Stefan L.M., Anton E.D., Berger D., Matei C., Negreanu-Pirjol T., Moldovan L. (2019). Physicochemical and Biological Properties of Gelatin Extracted from Marine Snail Rapana venosa. Mar. Drugs.

[86] Thandapani G., Prasad S., Sudha P.N., Sukumaran A. (2017). Size optimization and in vitro biocompatibility studies of chitosan nanoparticles. Int. J. Biol. Macromol..

[87] Ahmed H., Hashim A. (2020). Structure, Optical, Electronic and Chemical Characteristics of Novel (PVA-CoO) Structure Doped with Silicon Carbide. Silicon.

[88] Kodama Y., Kuramoto H., Mieda Y., Muro T., Nakagawa H., Kurosaki T., Sakaguchi M., Nakamura T., Kitahara T., Sasaki H. (2016). Application of biodegradable dendrigraft poly-l-lysine to a small interfering RNA delivery system. J. Drug Target..

[89] Song R., Murphy M., Li C., Ting K., Soo C., Zheng Z. (2018). Current development of biodegradable polymeric materials for biomedical applications. Drug Des. Dev. Ther..

[90] Ekici S., Ilgin P., Butun S., Sahiner N. (2011). Hyaluronic acid hydrogel particles with tunable charges as potential drug delivery devices. Carbohydr. Polym..

[91] Ilkhanizadeh S., Khalafy J., Dekamin M.G. (2019). Sodium alginate: A biopolymeric catalyst for the synthesis of novel and known polysubstituted pyrano[3,2-c]chromenes. Int. J. Biol. Macromol..

[92] Huang P., Lin J., Li W., Rong P., Wang Z., Wang S., Wang X., Sun X., Aronova M., Niu G. (2013). Biodegradable Gold Nanovesicles with an Ultrastrong Plasmonic Coupling Effect for Photoacoustic Imaging and Photothermal Therapy. Angew. Chem..

[93] Batrakova E.V., Bronich T.K., Vetro J.A., Kabanov A.V. (2006). Polymer Micelles as Drug Carriers. Nanoparticulates as Drug Carriers.

[94] Ak H.P.S., Saurabh C.K., Nurul Fazita M.R., Syakir M.I., Davoudpour Y., Rafatullah M., Abdullah C.K., Haafiz M.M.K., Dungani R. (2016). A review on chitosan-cellulose blends and nanocellulose reinforced chitosan biocomposites: Properties and their applications. Carbohydr. Polym..

[95] Sharifianjazi F., Khaksar S., Esmaeilkhanian A., Bazli L., Eskandarinezhad S., Salahshour P., Sadeghi F., Rostamnia S., Vahdat S.M. (2022). Advancements in Fabrication and Application of Chitosan Composites in Implants and Dentistry: A Review. Biomolecules.

[96] Su S., Kang P.M. (2020). Systemic Review of Biodegradable Nanomaterials in Nanomedicine. Nanomaterials.

[97] Laine C., Harlin A., Hartman J., Hyvärinen S., Kammiovirta K., Krogerus B., Pajari H., Rautkoski H., Setälä H., Sievänen J. (2013). Hydroxyalkylated xylans–Their synthesis and application in coatings for packaging and paper. Ind. Crops Prod..

[98] Moraga G., Talens P., Moraga M.J., Martínez-Navarrete N. (2011). Implication of water activity and glass transition on the mechanical and optical properties of freeze-dried apple and banana slices. J. Food Eng..

[99] Bankura K.P., Maity D., Mollick M.M.R., Mondal D., Bhowmick B., Bain M.K., Chakraborty A., Sarkar J., Acharya K., Chattopadhyay D. (2012). Synthesis, characterization and antimicrobial activity of dextran stabilized silver nanoparticles in aqueous medium. Carbohydr. Polym..

[100] Kanmani P., Lim S.T. (2013). Synthesis and characterization of pullulan-mediated silver nanoparticles and its antimicrobial activities. Carbohydr. Polym..

[101] Liu Y., Chen S., Zhong L., Wu G. (2009). Preparation of high-stable silver nanoparticle dispersion by using sodium alginate as a stabilizer under gamma radiation. Radiat. Phys. Chem..

[102] Islam M.S., Yeum J.H. (2013). Electrospun pullulan/poly(vinyl alcohol)/silver hybrid nanofibers: Preparation and property characterization for antibacterial activity. Colloids Surf. Physicochem. Eng. Asp..

[103] Venkatesan J., Singh S., Anil S., Kim S.-K., Shim M. (2018). Preparation, Characterization and Biological Applications of Biosynthesized Silver Nanoparticles with Chitosan-Fucoidan Coating. Molecules.

[104] Youssef A.M., Abou-Yousef H., El-Sayed S.M., Kamel S. (2015). Mechanical and antibacterial properties of novel high performance chitosan/nanocomposite films. Int. J. Biol. Macromol..

[105] Kraśniewska K., Galus S., Gniewosz M. (2020). Biopolymers-Based Materials Containing Silver Nanoparticles as Active Packaging for Food Applications–A Review. Int. J. Mol. Sci..

[106] Chitrakar B., Zhang M., Bhandari B. (2021). Improvement strategies of food supply chain through novel food processing technologies during COVID-19 pandemic. Food Control.

[107] Ghosh M., Singh A.K. (2022). Potential of engineered nanostructured biopolymer based coatings for perishable fruits with Coronavirus safety perspectives. Prog. Org. Coat..

[108] Calderón-Oliver M., Ponce-Alquicira E. (2018). Fruits: A Source of Polyphenols and Health Benefits. Natural and Artificial Flavoring Agents and Food Dyes.

[109] Mehany T., Khalifa I., Barakat H., Althwab S.A., Alharbi Y.M., El-Sohaimy S. (2021). Polyphenols as promising biologically active substances for preventing SARS-CoV-2: A review with research evidence and underlying mechanisms. Food Biosci..

[110] Paraiso I.L., Revel J.S., Stevens J.F. (2020). Potential use of polyphenols in the battle against COVID-19. Curr. Opin. Food Sci..

[111] Mahmoud Y.A.G., El-Naggar M.E., Abdel-Megeed A., El-Newehy M. (2021). Recent Advancements in Microbial Polysaccharides: Synthesis and Applications. Polymers.

[112] Baird J.K., Sandford P.A., Cottrell I.W. (1983). Industrial Applications of Some New Microbial Polysaccharides. Biotechnology.

[113] Aluwihare L.I., Repeta D.J. (1999). A comparison of the chemical characteristics of oceanic DOM and extracellular DOM produced by marine algae. Mar. Ecol. Prog. Ser..

[114] Kimmel S.A., Roberts R.F., Ziegler G.R. (1998). Optimization of Exopolysaccharide Production by Lactobacillus delbrueckii subsp. bulgaricus RR Grown in a Semidefined Medium. Appl. Environ. Microbiol..

[115] Nwodo U., Green E., Okoh A. (2012). Bacterial Exopolysaccharides: Functionality and Prospects. Int. J. Mol. Sci..

[116] Özcan E., Öner E.T. (2015). Microbial Production of Extracellular Polysaccharides from Biomass Sources. Polysaccharides.

[117] Višić K., Pušić T., Čurlin M. (2021). Carboxymethyl Cellulose and Carboxymethyl Starch as Surface Modifiers and Greying Inhibitors in Washing of Cotton Fabrics. Polymers.

[118] Mirzaei M., Alimi M., Shokoohi S., Golchoobi L. (2018). Synergistic interactions between konjac-mannan and xanthan/tragacanth gums in tomato ketchup: Physical, rheological, and textural properties. J. Texture Stud..

[119] Zhao Y., Sun H., Yang B., Weng Y. (2020). Hemicellulose-Based Film: Potential Green Films for Food Packaging. Polymers.

[120] Thakur B.R., Singh R.K., Handa A.K., Rao M.A. (2009). Chemistry and uses of pectin—A review. Crit. Rev. Food Sci. Nutr..

[121] Santana Á.L., Meireles M.A.A. (2014). New Starches are the Trend for Industry Applications: A Review. Food Public Health.

[122] BeMiller J.N. (2019). Xanthan. Carbohydrate Chemistry for Food Scientists.

[123] Singh R.S., Kaur N., Kennedy J.F. (2019). Pullulan production from agro-industrial waste and its applications in food industry: A review. Carbohydr. Polym..

[124] Puscaselu R.G., Lobiuc A., Dimian M., Covasa M. (2020). Alginate: From Food Industry to Biomedical Applications and Management of Metabolic Disorders. Polymers.

[125] Ruelas-Chacon X., Aguilar-González A., de la Luz Reyes-Vega M., Peralta-Rodríguez R.D., Corona-Flores J., Rebolloso-Padilla O.N., Aguilera-Carbo A.F. (2020). Bioactive Protecting Coating of Guar Gum with Thyme Oil to Extend Shelf Life of Tilapia (*Oreoschromis niloticus*) Fillets. Polymers.

[126] BeMiller J.N. (2019). Gum Arabic and Other Exudate Gums. Carbohydrate Chemistry for Food Scientists.

[127] Mostafavi F.S., Zaeim D. (2020). Agar-based edible films for food packaging applications—A review. Int. J. Biol. Macromol..

[128] Omoto T., Uno Y., Asai I. (1999). The latest technologies for the application of gellan gum. Physical Chemistry and Industrial Application of Gellan Gum.

[129] Mudgil D., Barak S. (2019). Classification, Technological Properties, and Sustainable Sources. Dietary Fiber: Properties, Recovery, and Applications.

[130] Peltzer M., Delgado J.F., Salvay A.G., Wagner J.R. (2018). β-Glucan, a Promising Polysaccharide for Bio-based Films Developments for Food Contact Materials and Medical Applications. Curr. Org. Chem..

[131] Felton L.A. (2013). Mechanisms of polymeric film formation. Int. J. Pharm..

[132] Thammakiti S., Suphantharika M., Phaesuwan T., Verduyn C. (2004). Preparation of spent brewer’s yeast beta-glucans for potential applications in the food industry. Int. J. Food Sci. Technol..

[133] Konusova V., Vorbeychikov E., Shamtsyan M. (2021). Potential role of mushroom beta-glucans in immunity and inflammation in viral infections and COVID-19. J. Food Bioact..

[134] Vetvicka V., Vetvickova J. (2011). β(1-3)-D-glucan affects adipogenesis, wound healing and inflammation. Orient. Pharm. Exp. Med..

[135] Pisanello D., Caruso G. (2018). Novel Foods in the European Union.

[136] Zhu F., Du B., Xu B. (2016). A critical review on production and industrial applications of beta-glucans. Food Hydrocoll..

[137] Henrion M., Francey C., Lê K.-A., Lamothe L. (2019). Cereal B-Glucans: The Impact of Processing and How It Affects Physiological Responses. Nutrients.

[138] Ludwicka K., Kaczmarek M., Białkowska A. (2020). Bacterial Nanocellulose—A Biobased Polymer for Active and Intelligent Food Packaging Applications: Recent Advances and Developments. Polymers.

[139] Huijbrechts A.M. (2008). Multifunctional Starch Derivatives: Synthesis, Characterization and Properties.

[140] Stephen A.M., Phillips G.O. (2016). Food Polysaccharides and Their Applications.

[141] Eerlingen R.C., Delcour J.A. (1995). Formation, analysis, structure and properties of type III enzyme resistant starch. J. Cereal Sci..

[142] Chen S., Chen Y., Wang Z., Chen H., Fan D. (2021). Renewable bio-based adhesive fabricated from a novel biopolymer and soy protein. RSC Adv..

[143] Williams P.A., Phillips G.O. (2021). Introduction to food hydrocolloids. Handbook of Hydrocolloids.

[144] Wilderjans E., Luyts A., Goesaert H., Brijs K., Delcour J.A. (2010). A model approach to starch and protein functionality in a pound cake system. Food Chem..

[145] Baranwal K., Dwivedi L.M., Shehala, Singh V. (2018). Guar gum mediated synthesis of NiO nanoparticles: An efficient catalyst for reduction of nitroarenes with sodium borohydride. Int. J. Biol. Macromol..

[146] Miyazawa T., Funazukuri T. (2006). Noncatalytic hydrolysis of guar gum under hydrothermal conditions. Carbohydr. Res..

[147] Dea I.C.M., Morrison A. (1975). Chemistry and Interactions of Seed Galactomannans. Advances in Carbohydrate Chemistry and Biochemistry.

[148] Tripathy S., Das M.K. (2013). Guar Gum: Present Status and Applications. J. Pharm. Sci. Innov..

[149] Wielinga W.C. (2009). Galactomannans. Handbook of Hydrocolloids.

[150] Shen Y., Babu K.S., Amamcharla J., Li Y. (2022). Emulsifying properties of pea protein/guar gum conjugates and mayonnaise application. Int. J. Food Sci. Technol..

[151] Bhat V.G., Narasagoudr S.S., Masti S.P., Chougale R.B., Vantamuri A.B., Kasai D. (2022). Development and evaluation of Moringa extract incorporated Chitosan/Guar gum/Poly (vinyl alcohol) active films for food packaging applications. Int. J. Biol. Macromol..

[152] Singh D., Viswakarma P., Kumar B. (2021). A review of guar gum processing, properties, and its food applications. Asian J. Multidimens. Res..

[153] Goemaere O., De Ketelaere B., Hanskens J., Masijn Q., Pérez Santaescolastica C., Fraeye I. (2021). Comparison of the Technological Application Potential of Functional Ingredients for the Meat Industry Based upon a Novel Fast Screening Tool. Foods.

[154] Adimule V., Kerur S.S., Chinnam S., Yallur B.C., Nandi S.S. (2022). Guar Gum and its Nanocomposites as Prospective Materials for Miscellaneous Applications: A Short Review. Top. Catal..

[155] Hesarinejad M.A., Lorenzo J.M., Rafe A. (2021). Influence of gelatin/guar gum mixture on the rheological and textural properties of restructured ricotta cheese. Carbohydr. Polym. Technol. Appl..

[156] Deshmukh R.K., Akhila K., Ramakanth D., Gaikwad K.K. (2022). Guar gum/carboxymethyl cellulose based antioxidant film incorporated with halloysite nanotubes and litchi shell waste extract for active packaging. Int. J. Biol. Macromol..

[157] Lee Y., Chang Y.H. (2016). Influence of guar gum addition on physicochemical, microbial, rheological and sensory properties of stirred yoghurt. Int. J. Dairy Technol..

[158] Mudgil D., Barak S., Khatkar B.S. (2011). Guar gum: Processing, properties and food applications—A Review. J. Food Sci. Technol..

[159] Diaz M., Ladero V., del Rio B., Redruello B., Fernández M., Martin M.C., Alvarez M.A. (2016). Biofilm-Forming Capacity in Biogenic Amine-Producing Bacteria Isolated from Dairy Products. Front. Microbiol..

[160] Baranwal K., Dwivedi L.M., Siddique S., Tiwari S., Singh V. (2020). Chitosan Grown Copper Doped Nickel Oxide Nanoparticles: An Excellent Catalyst for Reduction of Nitroarenes. J. Cluster Sci..

[161] Li B., Elango J., Wu W. (2020). Recent Advancement of Molecular Structure and Biomaterial Function of Chitosan from Marine Organisms for Pharmaceutical and Nutraceutical Application. Appl. Sci..

[162] Karthik R., Manigandan V., Saravanan R., Rajesh R.P., Chandrika B. (2016). Structural characterization and in vitro biomedical activities of sulfated chitosan from Sepia pharaonis. Int. J. Biol. Macromol..

[163] Wan A., Xu Q., Sun Y., Li H. (2013). Antioxidant Activity of High Molecular Weight Chitosan and N,O-Quaternized Chitosans. J. Agric. Food Chem..

[164] Manigandan V., Karthik R., Ramachandran S., Rajagopal S. (2018). Chitosan Applications in Food Industry. Biopolymers for Food Design.

[165] EUBIA Biopolymers. https://www.eubia.org/cms/wiki-biomass/bio-based-products/biopolymers/.

[166] Biodegradable Plastics Market by Type (PLA, Starch Blends, PHA, Biodegradable Polyesters), End Use Industry (Packaging, Consumer Goods, Textile, Agriculture & Horticulture), and Region (APAC, Europe, North America & RoW)–Global Forecast to 2026. https://www.marketsandmarkets.com/Market-Reports/biodegradable-plastics-93.html.

[167] A.R.C. Biopolymers Market–Forecast (2022–2027). https://www.industryarc.com/Report/11739/biopolymers-market.html.

